# THE IMPACT OF THE PROGRAM OF ALL-INCLUSIVE CARE FOR THE ELDERLY (PACE) CLOSURE ON SOCIAL ISOLATION AND LONELINESS

**DOI:** 10.1093/geroni/igad104.2737

**Published:** 2023-12-21

**Authors:** Barbara Dabrowski, Elizabeth Punke, Abby Teply, Katy Richardson, Christine McKibbin, Catherine Carrico

**Affiliations:** University of Wyoming, Laramie, Wyoming, United States; University of Wyoming, Laramie, Wyoming, United States; University of Wyoming, Laramie, Wyoming, United States; University of Wyoming, Laramie, Wyoming, United States; University of Wyoming, Laramie, Wyoming, United States; University of Wyoming, Laramie, Wyoming, United States

## Abstract

Social isolation and loneliness are public health problems seen in late adulthood that are associated with health risks (e.g., premature mortality, depression, and suicidality) and these issues are even more common among older adults with chronic illnesses (Nicholson, 2012). The Program of All-Inclusive Care for the Elderly (PACE) was developed to serve medical and behavioral needs and provide opportunities for social engagement for older adults with chronic illnesses. Budget cuts shut down Wyoming’s PACE program. This study aimed to (1) examine the impact of this closure on social isolation and loneliness, (2) identify how individuals have adapted to the loss of services that address social isolation, and (3) identify needs and preferences for future intervention to address social isolation. A concurrent mixed-methods design was used to examine these aims. Participants (n = 12; M = 74 years old, SD = 9.5) were predominantly White (n = 8, 66%), female (n = 6, 50%), and lived alone (n = 6, 50%). Caregivers of participants (n = 5; M = 63 years old, SD = 11) were predominantly Hispanic, Latino, or of Spanish origin (n = 3, 60%), female (n = 4, 80%), and lived with a partner (n = 4, 80%). Four central themes emerged: social isolation, emotional concerns, transportation loss, and care loss. Data showed decreases in social support and increases in loneliness. Results suggest programs like PACE greatly benefit older adults. Future directions may include developing community-based interventions to address service needs and social isolation concerns in Wyoming.

